# Antagonistic effects of endostatin-vascular endothelial growth inhibitor chimeric recombinant adenovirus on homocysteine-induced vascular endothelial cells injury in vitro and in vivo

**DOI:** 10.1097/MD.0000000000005197

**Published:** 2016-11-04

**Authors:** Zhen-Tian Cui, Jian-Ping Liu, Jian-Min Yao

**Affiliations:** a Cardiovascular Surgery, PLA Army General Hospital; b Navy Technical Investigation Bureau Health Team, Chinese People's Liberation Army, Beijing, P. R. China.

**Keywords:** antagonistic effect, endostatin, homocysteine, recombinant adenovirus, vascular endothelial cells injury, vascular endothelial growth inhibitor

## Abstract

**Background:**

The study is inclined to investigate the antagonistic effects of endostatin-vascular endothelial growth inhibitor chimeric recombinant adenovirus (Ad-hENDO-VEGI) on homocysteine (Hcy)-induced vascular endothelial cells (VECs) injury in vitro and in vivo.

**Methods:**

Human VECs cell line ECV304 was selected and infected with Ad-hENDO-VEGI. The LDH leakage, SOD activity, and MDA levels were measured by the automatic biochemical analyzer. Cell survival rate was counted by Trypanblau dying. The TNF-α and MCP-1 protein expressions were detected by ELISA assay. The protein expressions of fusion protein of Ad-hENDO-VEGI, nuclear factor kappa B p65 (NF-kappa B p65), and NF-kappa B inhibitor alpha (I-kappa B-α) were detected by Western blotting. A rat model of hyper-homocysteinemia was constructed. Thirty-six Wistar rats were randomly divided into 3 groups: the control group, the model group, and the Ad-hENDO-VEGI group. Serum Hcy levels in rats were measured with enzymatic cycling method. Endothelial vasodilation function was evaluated with the treatment of sodium nitroprusside and acetylcholine.

**Results:**

After Ad-hENDO-VEGI infection, high expressions (41 kD) of fusion proteins in ECV304 cells were observed. The injury severity of Hcy on ECV304 cells had a dose-dependent manner, and the injury reached a steady stage at 1.0 mmol/L. Thus, 1.0 mmol/L Hcy was selected for further experiments. With an increase of Ad-hENDO-VEGI in ECV304 cells after Hcy treatment, LDH leakage, MDA, TNF-α, MCP-1, and nuclear NF-kappa B p65 protein expression were gradually decreased, and cell survival rate, SOD activity, and I-kappa B-α protein expression were gradually increased. Compared with the control group, the model group had a higher Hcy level and attenuated vasodilator response. The Ad-hENDO-VEGI group exhibited a lower Hcy level and enhanced vasodilator response than the model group.

**Conclusion:**

These results indicated that Ad-hENDO-VEGI could down-regulate NF-kappa B p65 expression and suppress inflammatory response, thereby alleviating Hcy-induced VECs injury.

## 1 Introduction

Vascular endothelium is a layer of single cells lining on blood vessels, which serves as a multifunctional interface that exhibits a compelling phenotypic plasticity essential for maintenance of vascular homeostasis.^[1]^ The vascular endothelial cells (VECs) are crucial in the process of inflammatory and immune responses and also can trigger thrombosis and regulate vasomotor tone as well as vascular permeability.^[2]^ Dysfunction of the endothelium is reported to exert a significant pathogenic influence in cardiovascular-related diseases, such as atherosclerosis and its possible consequences: strokes and heart attacks.^[3]^ Santulli pointed out that the injury of endothelial cells (ECs) plays a fundamental role in atherogenesis, and EC repair may be of significance to prevent restenosis, and in-stent thrombosis as well.^[4]^ The improvement of EC functions and reendothelialization has been proved to be a potential therapy for the inhibition of restenosis, inflammation, as well as neoatherosclerosis. A microRNA-based approach with target sequences for EC-specific miR-126-3p at the 3′ end demonstrated this finding.^[5]^ Another microRNA (miR-223) was clearly shown to suppress endothelial inflammation and reactivity so as to prevent atherosclerosis-related leukocyte infiltration and inflammation.^[6]^ Homocysteine (Hcy) is an amino acid that biologically functions in methionine metabolism.^[7]^ Hcy induces endothelial dysfunction through multiple mechanisms one of which is endoplasmic reticulum (ER) stress. Severe ER stress induced by Hcy causes cell apoptosis, metabolic disorders, inflammation, and so on.^[8]^ An elevated level of total homocysteine (tHcy) in blood can serve as a strong risk factor for atherosclerotic vascular disease in the coronary, cerebral, and peripheral vessels, and for arterial and venous thromboembolism as well as cognitive decline, dementia, and Alzheimer disease.^[9]^ Thus, to explore the possible mechanism and molecular of protecting VECs from Hcy-induced injury is essential for the treatment of vascular diseases.

Endostatin, a 20 kD proteolytic fragment of collagenous type XVIII, has been well characterized as an effective agent for antiangiogenesis.^[10]^ It specifically suppresses angiogenesis through the inhibition of proliferation and migration, and the promotion of apoptosis in endothelium cells.^[11]^ Vascular endothelial growth inhibitor (VEGI) is an important member of tumor necrosis factor (TNF) superfamily that is produced mainly by endothelial cells.^[10]^ As VEGI could indirectly suppress proliferation of endothelial cell and angiopoiesis, recombinant human vascular endothelial growth inhibitor (rhVEGI) was found to be a novel inhibitor of endothelial cell proliferation.^[12]^ Moreover, Li et al^[10]^ stated that overexpression of endostatin-vascular endothelial growth inhibitor (ENDO-VEGI) fusion protein showed an inhibitory effect on angiogenesis. Therefore, we speculate combination of endostatin with VEGI might be a promising target for Hcy-induced VECs injury. Although several exogenous factors, such as genistein and theaflavins, have been shown to play important role in inhibiting Hcy-induced VECs injury, and protecting ECs from inflammatory injury or secretory dysfunction.^[13,14]^ There are few studies connecting recombinant molecule of ENDO-VEGI and its possible targeting Hcy in the treatment for VECs injury. In the present study, we aim to explore the antagonistic effects of endostatin-vascular endothelial growth inhibitor chimeric recombinant adenovirus (Ad-hENDO-VEGI) on Hcy-induced VECs injury in vitro and in vivo.

## 2 Materials and methods

### 2.1 Cell culture and grouping

The Roswell Park Memorial Institute-1640 (RPMI-1640) medium containing 10% fetal calf serum (FCS) was used for culture. After 100 mg/L penicillin and 100 mg/L streptomycin (Sigma-Aldrich Chemical Company, St Louis, MO) was added into the medium, human vascular endothelial cells (VECs) were cultured at 37°C, with 5% CO_2_ and saturated humidity. When cells reached 80% confluency, cells were subcultured. After cells were rinsed with phosphate buffer saline (PBS) 2 times, they were digested with 1 mL 0.25% trypsin, and terminated by being placed into 10% FCS medium when cells turned round with lacuna. Then the single-cell suspension was prepared and separately bottled with supplemented medium. Cells were lightly shaken and cultured in an incubator. The confluent cells were inoculated in a 24-well culture plate at 5 × 10^4^ cells per well, and then were incubated in a 5% CO_2_ incubator (37°C) for 48 hours, followed by another 24-hour incubation in a serum-free medium. Injected with different concentrations of Hcy, cells were divided into 5 groups: blank group (culture medium without any treatment); 0.1 mmol/L Hcy group (0.1 mmol/L Hcy added into medium); 0.5 mmol/L Hcy group (0.5 mmol/L Hcy added into medium); 1.0 mmol/L Hcy group (1.0 mmol/L Hcy added into medium); 2.0 mmol/L Hcy group (2.0 mmol/L Hcy added into medium). After 6-hour culture, culture medium and cells were collected to evaluate the best Hcy concentration inducing VECs injury. This Hcy concentration was used for the later experiments. Furthermore, with the administration of recombinant adenovirus, experimental cells were divided into 7 groups: the blank group; the Hcy group; the Hcy + 50 μL Ad-hENDO-VEGI group; the Hcy + 100 μL Ad-hENDO-VEGI group; the Hcy + 200 μL Ad-hENDO-VEGI group; the Hcy + 400 μL Ad-hENDO-VEGI group; the Hcy + 400 μL Ad-LacZ group. After cells in these 7 groups were cultured at 37°C for 72 h, medium and cells were collected for later experiments. Homocysteine (Hcy) used in this study was purchased from Sigma-Aldrich Chemical Company.

### 2.2 Efficiency of infection with adenovirus vector

Endostatin-vascular endothelial growth inhibitor recombinant adenovirus (Ad-hENDO-VEGI) or AdLacZ (50 μL) was infected into 293 cells. After the addition of 150 μL complete medium and 3-day culture, cells and supernatant presenting cytopathic effect (CPE) were separately collected, followed by 3 cycles of freezing at −70°C and thawing at 37°C. Then the filtrate (crude recombinant adenovirus) was obtained with 0.45 um filtration membrane, and was reserved at −70°C for later experiments. Titers of 2 recombinant adenoviruses (Ad-hENDO-VEGI or AdLacZ) were determined by Tissue Culture Infective Dose 50 (TCID_50_). The ECV304 cells were inoculated in a 96-well plate at 1 × 10^4^ cells per well. Five hours after culture, AdLacZ viruses at multiplicity of infection (MOI) of 0.1, 5, 10, 20, 50, and 100 were respectively infected into the cells. After another 48 hours, X-gal was added and blue-stained cells were counted under the microscope.

### 2.3 Identification of Ad-hENDO-VEGI infected cells

The 293 and ECV304 cells were separately inoculated in a 96-well plate at a density of 1 × 10^6^ cells per well. And then after complete adherence within 5 hours, both 293 and ECV304 cells were separately infected with the Ad-hENDO-VEGI vector at MOI of 20 for 4 hours. The ECV304 cells were infected with empty AdLacZ vector for 4 hours. Then through repeated freezing (−70°C) and thawing (37°C) thrice, these cells were centrifuged at 10,000 r/min for 5 min at 4°C to collect supernatant. Subsequently, the supernatant (20 μL) was subjected to sodium dodecyl sulfate polyacrylamide gel electrophoresis (SDS-PAGE, 10%), and then transferred onto the nitrocellulose membrane (Millipore, Billerica, MA, USA). Blocked with skim milk powder (50 g/L) at room temperature for 1 hour, the membrane was added with diluted (1:100) rabbit anti-human vascular endothelial growth inhibitor (VEGI) polyclonal antibody (Cell Signaling Technology Inc, Beverly, MA, USA) and incubated overnight at 4°C. After another addition of diluted (1:500) goat anti-rabbit immunoglobulin G-horseradish peroxidase (IgG-HRP, Cell Signaling Technology Inc, Beverly, MA, USA), the membrane was continued to be incubated at 37°C for 1 hour. Diaminobenzidine (DAB) was used for coloration. After scanning or photography of films, the gel electrophoresis analysis system was applied to analyze the molecular weight and optical density (OD) of the target band. Human embryonic kidney 293 (HEK-293) cells and human umbilical vein endothelial cell line (ECV304) were purchased from Institute of Biochemistry and Cell Biology, Shanghai Institutes for Biological Sciences (SIBS), Chinese Academy of Sciences (CAS), Shanghai, China.

### 2.4 Trypanblau dying

The mixture (1:1) of 0.5% trypsin and 0.2% elhylene diamine tetraacetic acid (EDTA) was used to digest VECs. Then cells were rinsed by Hanks Balanced Salt Solution (HBSS, Sigma-Aldrich Chemical Company), and were diluted. Each 0.1-mL cell suspension was added with a small drop of 0.4% trypan blue (Sigma-Aldrich Chemical Company), staining for 3 to 5 min at room temperature. Then a drop of the cell suspension was dropped onto a glass slide overlayed with a cover glass. The cell morphology was observed under a high-power microscope. The dead cells were stained blue, swollen, and lustreless. The live cells were unstained and lustrous in normal shape. The dead and live cells were separately counted in 1000 cells to calculate the cell survival rate.

### 2.5 Detection of LDH leakage, SOD activity, and MDA levels

Automatic biochemical analyzer (Hitachi Ltd, Tokyo, Japan) was utilized to measure leakage of lactate dehydrogenase (LDH) in the supernate of the culture medium of each group. Besides, VECs were selected for preparing 1% homogenate with normal saline (NS). The total superoxide dismutase (SOD) activity and copper- and zinc-SOD (Cu-Zn SOD) activity in homogenate (30 μL in each sample) were detected by SOD reagent kit (Nanjing Institute of Jiancheng Biological Engineering, Nanjing, China). At the same time, another homogenate (10%) was prepared by the same way, and the malondialdehyde (MDA) level of homogenate (200 μL in each sample) was detected by MDA reagent kit (Nanjing Institute of Jiancheng Biological Engineering).

### 2.6 Enzyme-linked immunosorbent assay (ELISA)

Protein expressions of tumor necrosis factor-α (TNF-α) or monocyte chemoattractant protein-1 (MCP-1) in the VECs were detected with corresponding Elisa reagent kit (Shanghai Xitang Biological Technology Co, Ltd, Shanghai, China). Washing solution was prepared with the ELISA kit being placed at indoor temperature for 20 minutes. The coated plate was set with 10 standard wells including 2 blank control wells (no sample and reagent was added). Gradient dilution was performed in standard wells to obtain a standard curve. Subsequently, dilute sample was added into test wells of the coated plate. They were slightly shaken to be well mixed and sealed for a 30-minute culture at 37°C. After that, solution in wells was thrown away and then repeated 5 cycles (washing, 30 seconds; removing the washing solution), and dried in the end. Fifty microliters Elisa reagent was added to culture for 30 minutes at 37°C, after which solution in wells was thrown away and then repeated 5 cycles (washing, 30 seconds; throwing the washing solution), and dried in the end. Then 50 μL A color reagent and 50 μL B color reagent were added into each well successively, and well mixed. After they were incubated in darkness for 15 minutes at 37°C, 50 μL stop buffer was added to terminate developing. In 15 minutes, the OD value of each hole was determined at the wavelength of 450 nm, when that of the blank control well was set as zero. The standard curve was obtained with the concentration of standard sample as abscissa and the OD value as ordinate. The corresponding concentration of the sample was estimated by regression equation of standard curve according to the OD value.

### 2.7 Western blotting

Four-hour culture after infection, VECs were added into homogenizer, with the addition of lysis buffer and protease inhibitor (phenylmethanesulfonyl fluoride [PMSF]), to be homogenized for 30 minutes at 4°C. And then the supernate was obtained with centrifugation at 15,000 r/min. The target protein was extracted with a total protein extraction kit and nuclear and cytoplasmic protein extraction kit (Shanghai Sangon Biological Engineering Technology & Services Co, Ltd, Shanghai, China). The protein expression was detected by bicinchoninic acid (BCA) protein assay kit (Beyotime Institute of Biotechnology, Shanghai, China). Mixed with ×5 loading buffer (Beyotime Institute of Biotechnology) in samples, protein denaturation was conducted with 5-minute boiling at 100°C. Equal amounts of proteins were separated by SDS-PAGE (10%), electron-transferring onto nitrocellulose (NC) membrane (Amresco Inc, Cochran, OH, USA). The primary antibodies, nuclear factor kappa Bp (NF-kappa B, 1:500), and NF-kappa B inhibitor alpha (I-kappa B-α, 1:500) were diluted by Tris Buffered Saline With Tween (TBST, Beijing Biolab technology Co, Ltd). Sealed at room temperature with 5% skim milk, NC membrane was added with these 2 antibodies and stored overnight at 4°C. After 3 times washing of transfer membrane with TBST (5 min per time), corresponding second antibody was added for 2-hour reaction at 37°C. Followed by membrane washing, A color reagent and B color reagent (Promega Corporation, Madison, Wisconsin, USA) were mixed at a ratio of 1:1 to develop (1 minute) at room temperature. Then the membrane wrapped by plastic wrap was transferred into darkroom, with developing and fixing of exposed X-ray films. The results were analyzed by Gel-Pro analyzer 4.0 software. The NF-κB protein level was reflected by the ratio of gray value of the target protein and that of β-actin. The primary and second antibodies were purchased from Kangchen Biotechnology Company (Shanghai, China), decoloring shaker (ZD-9500) was from Hualida Experiment Equipment Co, Ltd, Taicang, China, and vertical trans-blot transfer was bought from Bio-Rad Laboratories Inc, CA (Mississauga, Ontario, Canada).

### 2.8 Construction of a rat model of hyper-homocysteinemia

The animal experiment was approved by the Ethics Committee of Biomedical Research Ethics Committee, SIBS, CAS. It was performed in conformity with the Helsinki Declaration. The Wistar male rats (n = 36; weights of 145 ± 32 g) were bought from Shanghai Institute of Material Medica, CAS. Wistar male rats (n = 36) were randomly divided into 3 groups including the control group (n = 12), the model group (n = 12), and the Ad-hENDO-VEGI group (n = 12). The rats in the control group (n = 12) were given food and water without restriction. The other 24 rats were given food (contained 2% methionine) and water for 8 weeks to build a rat model of hyper-homocysteinemia.

### 2.9 Detection of serum Hcy levels in experimental rats

After 8 weeks of feed, blood samples (0.5 mL blood each rat) of these rats were collected for detecting serum Hcy level. After citrate anticoagulation, these samples were immediately detected with Hitachi 7600 automatic biochemical analyzer (Hitachi Ltd, Tokyo, Japan). The serum Hcy level in experimental rats was measured by enzymatic cycling method (test kit provided by Beijing Strong Biotechnologies Inc, Beijing, China). If serum Hcy level >15 μmol/L observed in the blood sample, the model construction was successful. Then rats (n = 12) in the successful rat model were randomly selected, and injected with prepared cells (infected with Ad-hENDO-VEGI in the early experiment). Fresh blood samples of rats in each group were collected again for detecting serum Hcy level after Ad-hENDO-VEGI treatment, by cycling enzymatic method.

### 2.10 Evaluation of endothelial vasodilation function of experimental rats

These rats were anesthetized with intraperitoneal injection of 25% urethane (Sigma-Aldrich Chemical Company). Once they were completely anesthetized, the thoracic aortae of rats were rapidly taken out and stored at precooled Krebs–Henseleit solution for later arterial circle preparation (2–3 nm). These prepared circles were mixed with 5% CO_2_/95% O_2_, followed by a water bath at 37°C. Data were recorded and processed by BL-420F bio-functional experiment system (Chengdu Thai Union Biological Technology Co, Ltd, Sichuang, China). These arterial circles were given a tension of 1.0 g, and balanced for 60 minutes. After maximum amplitude of vasoconstriction was induced by phenylephrine (PE, 10 μmol/L, Sigma), endothelial vasodilation function was detected at different concentrations of sodium nitroprusside (10^−7^–10^−3^ mol/L, Beijing Double-Crane Pharmaceutical Co, Ltd, Beijing, China) or at different concentrations of acetylcholine (10^−6^–10^−3^ mol/L, Sigma-Aldrich Chemical Company).

### 2.11 Statistical analysis

SPSS 21.0 software (SPSS Inc, Chicago, IL) was employed for data analysis. Continuous data were shown as mean ± standard deviation. Besides, *t* test was used for the intergroup comparison, and one-way analysis of variance was used for comparison among multiple groups. Difference was considered to be statistically significant if *P* <0.05.

## 3 Results

### 3.1 Efficiency of infection with adenovirus vector

**Figure 1:**
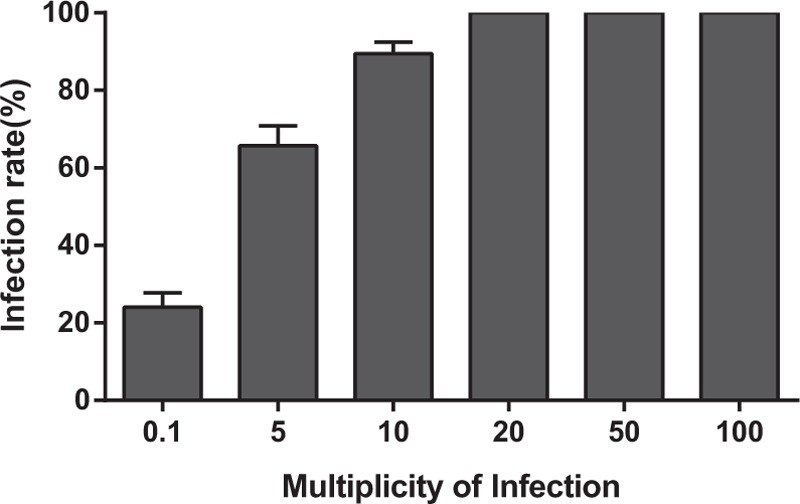
The efficiency of AdLacZ infection with different MOI. Note: MOI = multiplicity of infection.

The virus titer of the crude recombinant adenovirus was 1 × 10^12^ TCID_50_/L measured by TCID50. Blue-stained cells accounted for approximately 24.2% when the ECV304 cells were infected with AdLacZ at an MOI of 0.1. The percentage of blue-stained cells reached 100% when the MOI was increased to 20 (Fig. 1), suggesting that the crude recombinant adenovirus exhibited high efficiency of infection with the adenovirus vector.

### 3.2 Specific protein expression of Ad-hENDO-VEGI gene

**Figure 2:**
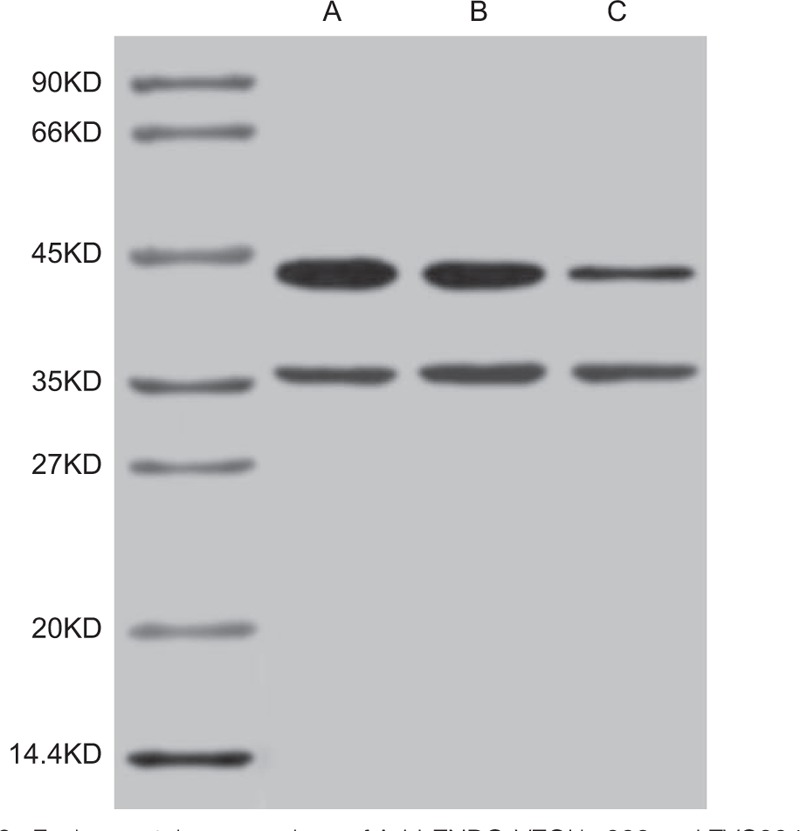
Fusion protein expressions of Ad-hENDO-VEGI in 293 and EVC304 cells detected by Western blotting. Note: A, the 293 cells infected with Ad-hENDO-VEGI. B, EVC304 cells infected with Ad-hENDO-VEGI. C, EVC304 cells infected with AdLacZ.

When the lysate of ECV304 cells were infected with empty Ad-LacZ vector, and the lysate of 2 cell groups (293 cells and ECV304 cells) were infected with Ad-hENDO-VEGI, the expressions of fusion proteins were detected by Western blotting. In accordance with the bands of fusion proteins shown as Fig. 2, higher expressions of fusion proteins in 293 and ECV304 cells (infected with Ad-hENDO-VEGI) were observed at 41KD than these in ECV304 cells (infected with empty Ad-LacZ vector). These results revealed the specific protein expression of *Ad-hENDO-VEGI* gene in several kinds of cells. This high protein expression might be mediated by the exogenous *Ad-hENDO-VEGI*.

### 3.3 Effects of different doses of Hcy on cell survival rate and LDH leakage in VECs

**Figure 3:**
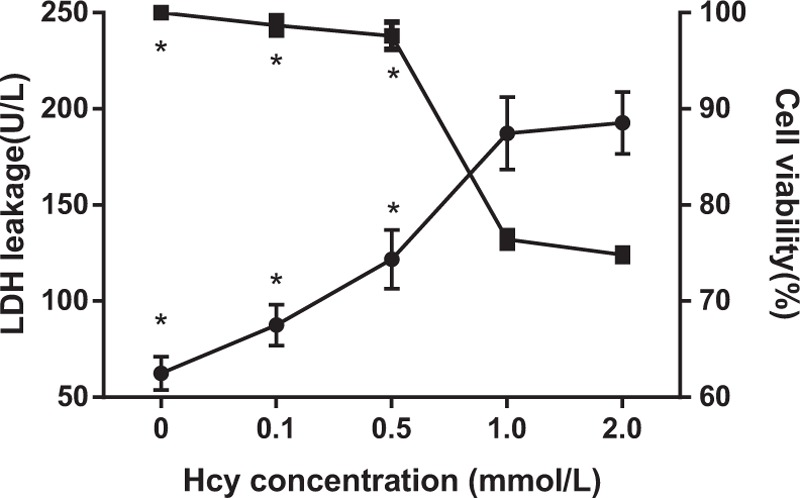
Effects of different doses of Hcy on LDH leakage and cell survival rate in VECs. Notes: Hcy = homocysteine, LDH = lactate dehydrogenase, VECs = vascular endothelial cells. ∗, *P* <0.05 compared with the 1 mmol/L Hcy group.

The LDH leakage (87.5 ± 10.6 U/L) and cell survival rate (98.2% ± 1.0%) in the 0.1 mmol/L Hcy group were not evidently different from these in the blank group (both *P* > 0.05) (Fig. 3). When compared with the 0.1 mmol/L Hcy group, LDH leakage significantly increased to (121.7 ± 15.3) U/L (*P* <0.05), while there was no significant difference in cell survival rate (97.7% ± 1.5%) (*P* > 0.05) in the 0.5 mmol/L Hcy group. In the 1.0 mmol/L Hcy group, the LDH leakage (187.2 ± 18.8 U/L) exhibited an increase of 124.8 U/L, and the cell survival rate (76.4% ± 0.8%) exhibited an decrease of 23.6% than these in the 0.5 mmol/L Hcy group (both *P* <0.05). When Hcy level was 2.0 mmol/L, both LDH leakage (192.7 ± 16.1 U/L) and cell survival rate (74.8% ± 0.7%) were not significantly different from these in the 0.1 mmol/L Hcy group (*P* > 0.05).

### 3.4 Effects of different doses of Hcy on MDA level and SOD activity in VECs

The MDA level was higher and the SOD activity was lower after Hcy treatment (0.1, 0.5, 1.0, 2 mmol/L) than these in the blank group (both *P* <0.05). Furthermore, the MDA level was increased while SOD activity was decreased with the increasing Hcy level (0.1, 0.5, 1.0 mmol/L) (all *P* <0.05). However, there was no significant difference of MDA level and SOD activity between Hcy 1.0 mmol/L group and Hcy 2.0 mmol/L group (both *P* >0.05) (Table 1).

**Table 1:**
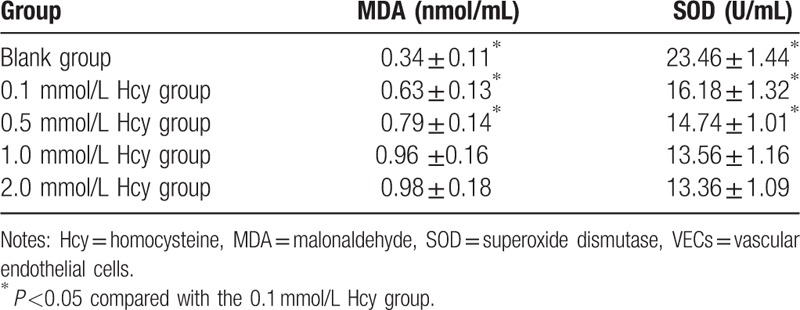
Effects of different doses of Hcy on MDA level and SOD activity in VECs.

### 3.5 Effects of different doses of Hcy on TNF-α and MCP-1 protein expressions in VECs

**Figure 4:**
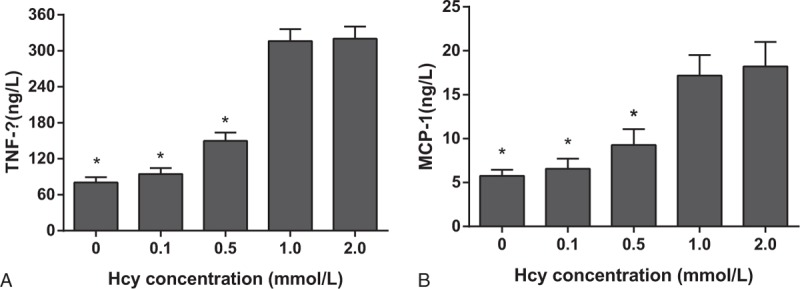
Effects of different doses of Hcy on TNF-α and MCP-1 expressions in VECs. Note: Hcy = homocysteine, MCP-1 = monocyte chemoattractant protein-1, TNF-α = tumor necrosis factor-alpha, VECs = vascular endothelial cells. ∗, *P* <0.05 compared with the 1 mmol/L Hcy group.

Compared with the blank group, TNF-α and MCP-1 protein expressions in the Hcy groups (0.1, 0.5, 1.0 mmol/L) were elevated as the level of Hcy raised. The 1.0 mmol/L Hcy group had significantly higher TNF-α and MCP-1 protein expressions than the 0.5 mmol/L Hcy group (*P* <0.05) (Fig. 4). But there was no significant difference of TNF-α and MCP-1 protein expressions between the 1.0 mmol/L Hcy group and the 2.0 mmol/L Hcy group (both *P* >0.05). Thus, the higher Hcy serum level induced a higher injury severity to VECs, which reached a steady state when Hcy level rose to 1.0 mmol/L. Thus, 1.0 mmol/L Hcy was selected for further experiments.

### 3.6 Effects of different concentrations of Ad-hENDO-VEGI on cell survival rate and LDH leakage in VECs

In Hcy + Ad-hENDO-VEGI groups (50, 100, 200, and 400 μL Ad-hENDO-VEGI), higher concentration of Ad-hENDO-VEGI caused gradual reductions of LDH leakage and an increase of cell survival rate (all *P* <0.05). Compared with the Hcy group (1.0 mmol/L Hcy), the cell survival rate showed an increasing tendency and the LDH leakage showed a declining tendency with the increased treatment of Ad-hENDO-VEGI (50, 100, 200, 400 uL) (all *P* <0.05). Both LDH leakage and cell survival rate in the Hcy + 400 uL Ad-hENDO-VEGI group were significantly lower than these in the blank group (both *P* <0.05). These 2 in the Hcy group were not significantly different from these in the Hcy+400 μL Ad-LacZ group (both *P* >0.05) (Table 2).

**Table 2:**
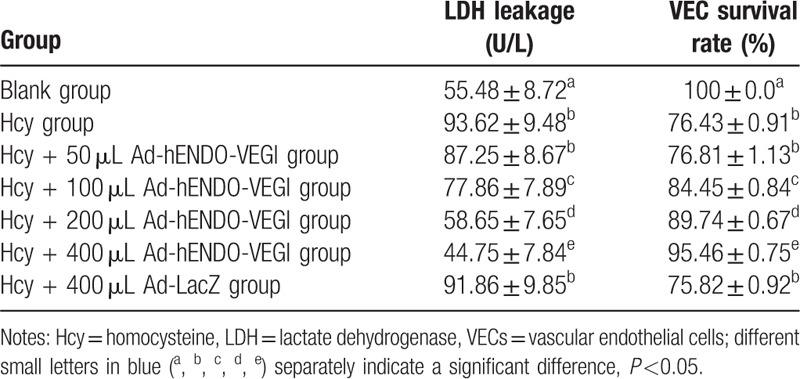
Effects of different concentrations of Ad-hENDO-VEGI on LDH leakage and cell survival rate of VECs.

### 3.7 Effects of different concentrations of Ad-hENDO-VEGI on SOD activity and MDA level in VECs

In Hcy + Ad-hENDO-VEGI groups (50, 100, 200, and 400 μL Ad-hENDO-VEGI), higher concentration of Ad-hENDO-VEGI caused gradual reductions of MDA level and an increase of SOD activity (all *P* <0.05). The MDA levels in the Hcy + Ad-hENDO-VEGI groups (50, 100, 200, 400 uL Ad-hENDO-VEGI) were separately much lower than that in the Hcy group (1.0 mmol/L Hcy), while the SOD activities were much higher (all *P* <0.05). Besides, both MDA level and SOD activity in the Hcy + 400 uL Ad-hENDO-VEGI group were significantly different from these in the blank group (both *P* <0.05). These 2 in the Hcy group were not significantly different from these in the Hcy + 400 μL Ad-LacZ group (*P* >0.05) (Table 3).

**Table 3:**
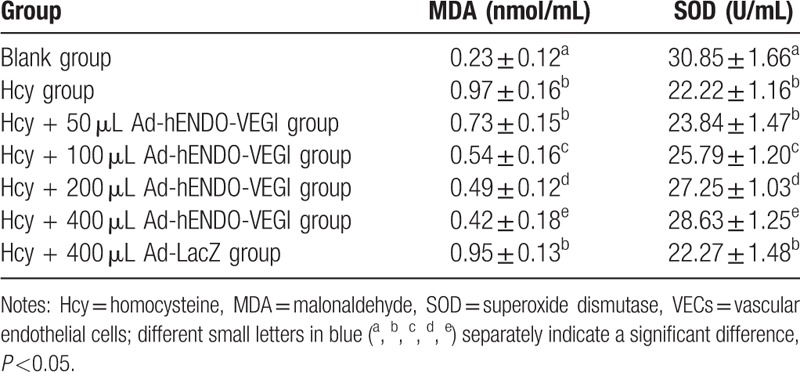
Effects of different concentrations of Ad-hENDO-VEGI on MDA level and SOD activity in VECs.

### 3.8 Effects of different concentrations of Ad-hENDO-VEGI on TNF-α and MCP-1 protein expressions in VECs

**Figure 5:**
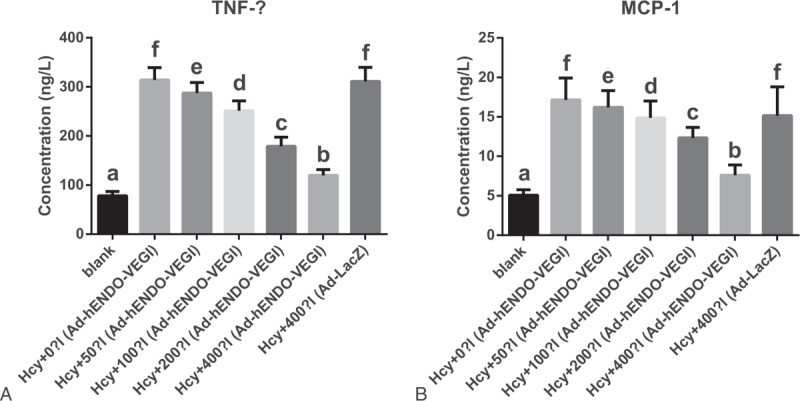
Effects of different concentrations of Ad-hENDO-VEGI on TNF-α and MCP-1 expressions in VECs. Note: Ad-hENDO-VEGI = endostatin-vascular endothelial growth inhibitor chimeric recombinant adenovirus, MCP-1 = monocyte chemoattractant protein-1, TNF-α = tumor necrosis factor-alpha, VECs = vascular endothelial cells; different letters (a, b, c, d, e, f) separately indicate a significant difference, *P* <0.05.

Compared with the blank group, other 6 groups (Hcy + 0 μL Ad-hENDO-VEGI group, Hcy + 50 μL Ad-hENDO-VEGI group, Hcy + 100 μL Ad-hENDO-VEGI group, Hcy + 200 μL Ad-hENDO-VEGI group, Hcy + 400 μL Ad-hENDO-VEGI group, and Hcy + 400 μL Ad-LacZ group) had high TNF-α and MCP-1 protein expressions (all *P* <0.05). Additionally, these 2 protein expressions were not evidently different between Hcy + 400 μL Ad-LacZ group and the Hcy group (0.1 mmol/L Hcy) (both *P* >0.05). After Ad-hENDO-VEGI treatment (50, 100, 200, and 400 μL), both TNF-α and MCP-1 protein expressions were reduced, showing a decreasing tendency as the volume of Ad-hENDO-VEGI increased (all *P* <0.05) (Fig. 5).

### 3.9 Effects of different concentrations of Ad-hENDO-VEGI on NF-kappa B p65 and I-kappa B protein expression in VECs

**Figure 6:**
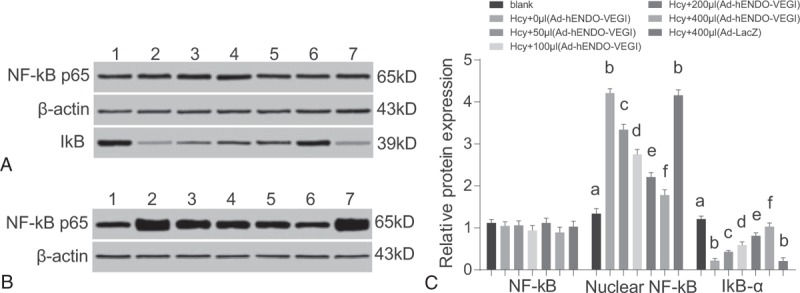
Effects of different concentrations of Ad-hENDO-VEGI on NF-kappa B p65 and I-kappa B-α protein expressions in VECs. Note: A, the NF-kappa Bp65 and I-kappa B protein expressions in VECs detected by Western blotting. B, The nuclear NF-kappa B p65 protein expression in VECs detected by Western blotting. C, Comparison of NF-kappa B p65 and I-kappa B-α protein expressions in VECs among 7 groups. Ad-hENDO-VEGI = endostatin-vascular endothelial growth inhibitor chimeric recombinant adenovirus, I-kappa B-α = NF-kappa B inhibitor alpha, NF-kappa B p65 = nuclear factor kappa B p65, VECs = vascular endothelial cells; different letters (a, b, c, d, e, f) separately indicate a significant difference, *P* <0.05.

Compared with the blank group, other 6 groups had higher nuclear NF-kappa B p65 protein expressions (all *P* <0.05). And the difference of NF-kappa B p65 protein expression between the Hcy group (0.1 mmol/L Hcy) and Hcy + 400 μL Ad-LacZ group exhibited no statistical significance (*P* >0.05). After Ad-hENDO-VEGI treatment (50, 100, 200, and 400 μL), the total protein expression of NF-kappa B p65 showed no much changes. However, the I-kappa B-α protein expression was gradually elevated and the nuclear NF-kappa B p65 protein expression was gradually down-regulated with the increasing volume of Ad-hENDO-VEGI (all *P* <0.05) (Fig. 6).

### 3.10 Comparison of serum Hcy levels in rats among the control, model, and Ad-hENDO-VEGI groups

Hcy levels in rats of both the model group and the Ad-hENDO-VEGI group were higher than that in the control group (both *P* <0.05). But the Hcy level in rats of the Ad-hENDO-VEGI group was lower than that in the model group (*P* <0.05). The Hcy level in rats of the model group was over 15 μmol/L (Table 4).

**Table 4:**
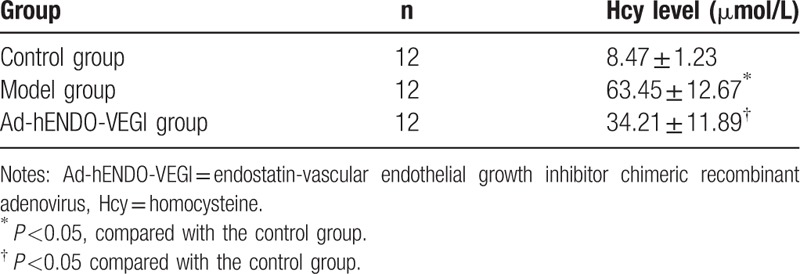
Comparison of serum Hcy levels in rats among the control, model, and Ad-hENDO-VEGI groups.

### 3.11 Comparison of vasodilator response in rats among the control, model, and Ad-hENDO-VEGI groups

Compared with the control group, the vasodilator response to different concentrations of sodium nitroprusside (10^−7^–10^−3^ mol/L) in the model group was attenuated, and the vasodilator response to different concentrations of acetylcholine (10^−6^–10^−3^ mol/L) was attenuated as well (both *P* <0.05). But these were enhanced in Ad-hENDO-VEGI group compared with the model group (both *P* <0.05) (Tables 5 and 6).

**Table 5:**
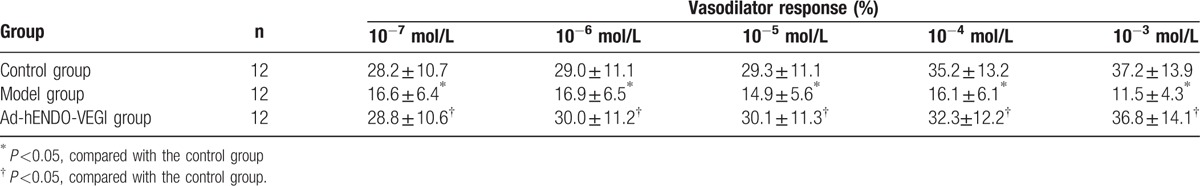
Comparison of vasodilator responses to different concentrations of sodium nitroprusside in rats among the control, model, and Ad-hENDO-VEGI groups.

**Table 6:**
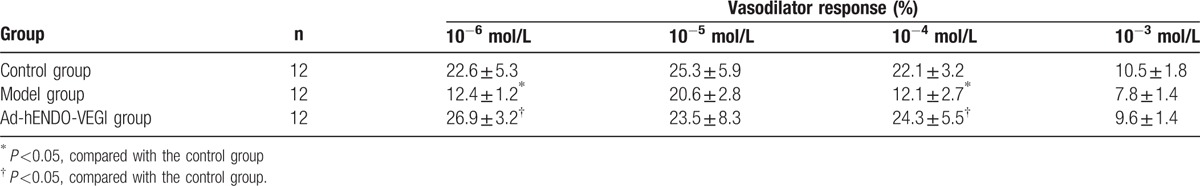
Comparison of vasodilator responses to different concentrations of acetylcholine in rats among the control, model, and Ad-hENDO-VEGI groups.

## 4 Discussion

It is known that Hcy can contribute to vascular dysfunction, and Hcy is involved in inducing vascular diseases such as atherosclerosis and thrombosis. This study aims to determine the effect of recombinant adenovirus Ad-hENDO-VEGI on the VECs injury due to Hcy, and probe into a potential treatment for Hcy-related vascular diseases.

Initially, this study demonstrated that as the increasing level of Hcy, LDH leakage, MDA level, TNF-α, and MCP-1 were gradually increased, and cell survival rate and SOD activity were declined. These results indicated that Hcy could cause an injury on VECs, which might be in a concentration-dependent manner. LDH leakage can be recognized as an indication of cell necrosis.^[15]^ Currently, it is recognized that Hcy impairs endothelial cells in a variety of ways. Lee et al^[16]^ found that Hcy caused cerebral endothelial cell death in murine by activation of Asm-ceramide pathway. Also, Xu et al^[17]^ reported that Hcy induced endothelial cell apoptosis possibly by a pathway involving caspase-3. It also has been reported that hyper-homocysteinemia impaired endothelium function due to loss of nitric oxide (NO) and decrease of endothelial relaxation.^[18]^ And Jia et al^[19]^ concluded that Hcy caused apoptosis of endothelial cells as a result of hypermethylation of dimethylarginine dimethylaminohydrolase 2 (DDAH2). Postea et al^[20]^ suggested that reactive oxygen species (ROS) (oxidative stress) caused by Hcy may induce a pro-inflammation in the vessel wall, which initiated and accelerated the development of atherosclerosis. Malondialdehyde (MDA), produced by lipid peroxidation, has been used as an indicator of oxidative stress.^[21]^ Moreover, ROS could be indirectly scavenged through endogenous antioxidant enzymes such as SOD.^[22]^ In this study, MDA level was increased and SOD activity was decreased after Hcy treatment in VECs. Therefore, Hcy might cause injury through stimulating ROS. Hung et al^[23]^ demonstrated that, via activating PKCβ, Hcy enhanced the expression of lectin-like oxidized low-density lipoprotein receptor-1 (LOX-1), which caused endothelium inflammation and significantly participated in the progression of atherosclerosis. Both TNF-α and MCP-1 have been considered major inflammatory cytokines.^[24]^ The protein expressions of these 2 indicators were observed to be elevated by Hcy in VECs. Therefore, Hcy might also induce injury via promoting inflammatory responses. And larger doses of Hcy could cause higher injury severity of VECs, which reached a steady stage at the level of 1.0 mmol/L Hcy.

To confirm whether Ad-hENDO-VEGI could affect Hcy in VECs, the relative factors mentioned in above were all evaluated after Ad-hENDO-VEGI treatment. In this study, when the concentration of recombinant adenovirus Ad-hENDO-VEGI increased, the cell survival rate and SOD activity were increased, and the MDA, TNF-α MCP-1, and LDH leakage were decreased. The results significantly indicated that the recombinant adenovirus Ad-hENDO-VEGI can effectively antagonize the impairment on human VECs caused by Hcy in a concentration-dependent manner. Inflammation and angiogenesis are often closely associated with each other in some biologic process such as response to injury, or function of wound healing in chronic inflammatory diseases such as atherosclerosis. Neovascularization may function to maintain vascular inflammation.^[25]^ Both ENDO and VEGI are recognized as endogenous angiostatin and can inhibit pathological angiogenesis. Ahmed et al^[26]^ showed that TNF-α and its stimulated-MCP-1 expressions in human vascular disease atherosclerosis were partially inhibited by JNKI-1 pathway, and VEGI was able to activate the signaling pathway JNKI-1.^[12]^ There is another study demonstrating that VEGI can depress capillary formation no matter what types of angiogenic stimuli.^[27]^ It has been reported that the overexpression of ENDO-VEGI fusion protein achieved a strong inhibition effect on angiogenesis.^[8]^ And Pan et al^[28]^ discovered that ENDO-VEGI chimeric recombinant adenovirus exhibited significant inhibiting efficacy on neovascularization. The former 2 studies revealed that recombinant adenovirus had an effect on angiogenesis which could further influence inflammatory responses. In line with these findings, our study revealed antagonistic effects of Ad-hENDO-VEGI through inhibiting the inflammatory responses. Nuclear factor kappa B (NF-κB) is known as a pro-inflammatory factor, and plays a crucial role in inflammatory and immune processes by regulating expressions of inflammatory genes such as TNF-α IL-6 and so on.^[29]^ The nuclear NF-kappa B p65 protein expression was decreased while Ikappa B-α (NF-kappa B inhibitor alpha) protein expression was significantly elevated after Ad-hENDO-VEGI treatment in the VECs with Hcy-induced injury. Meanwhile, the animal experiment showed the Hcy level was reduced by Ad-hENDO-VEGI in the Ad-hENDO-VEGI group. It suggested an improvement of endothelial vasodilation function after Ad-hENDO-VEGI treatment, further verifying our findings.

In conclusion, our study suggests that Ad-hENDO-VEGI could down-regulate NF-kappa B p65 expression and suppress inflammatory response, thereby alleviating Hcy-induced VECs injury. This finding would be hopeful for treatment of Hcy-related cardiovascular diseases. However, one of the limitations is lack of several experiments to confirm the specific mechanism. Further study on specific mechanisms of the antagonistic effects of fusion protein of ENDO and VEGI on Hcy-induced VECs injury should be performed in the future to provide better treatment for Hcy and its related diseases.

## Acknowledgments

We would like to express our gratitude to the reviewers for their helpful comments on this paper.
